# Pigs as Pets: Early Human Relations with the Sulawesi Warty Pig (*Sus celebensis*)

**DOI:** 10.3390/ani13010048

**Published:** 2022-12-22

**Authors:** Adam Brumm

**Affiliations:** Australian Research Centre for Human Evolution, Griffith University, Brisbane, QLD 4111, Australia; a.brumm@griffith.edu.au

**Keywords:** Sulawesi warty pigs, *Sus celebensis*, pig domestication, pet-keeping, Pleistocene

## Abstract

**Simple Summary:**

In the early 1980s, the late Colin Groves (1942–2017), a noted taxonomist and suid specialist, proposed that the Sulawesi warty pig (*Sus celebensis*), an endemic suid from the Indonesian island of Sulawesi, had been independently domesticated by a pre-Neolithic human population (that is, a non-sedentary foraging society) and translocated by watercraft to other islands in the region. This conflicts with two central premises in our understanding of the origins of animal domestication: (1) that the wolf was the only animal domesticated by hunter-gatherers prior to the Neolithic farming transition around 12,000–10,000 years ago; and (2) that the beginnings of pig domestication were inextricably tied to the advent of crop-raising and settled agrarian communities. This paper considers whether it is plausible to suggest that a non-sedentary population of hunter-gatherers could have domesticated a wild suid. It is proposed that pre-agricultural foragers could have established a close association with wild-living members of *S. celebensis* that was similar to the relationship of interspecies companionship that is purported to have existed between Late Pleistocene foragers and juvenile wolves, according to some, leading to the first domesticated dogs.

**Abstract:**

The Sulawesi warty pig (*S. celebensis*) is a wild and still-extant suid that is endemic to the Indonesian island of Sulawesi. It has long been theorised that *S. celebensis* was domesticated and/or deliberately introduced to other islands in Indonesia prior to the advent of the Neolithic farming transition in the region. Thus far, however, there has been no empirical support for this idea, nor have scientists critiqued the argument that *S. celebensis* was a pre-Neolithic domesticate in detail. Here, it is proposed that early foragers could have formed a relationship with *S. celebensis* that was similar in essence to the close association between Late Pleistocene foragers in Eurasia and the wild wolf ancestors of domestic dogs. That is, a longstanding practice of hunter-gatherers intensively socialising wild-caught *S. celebensis* piglets for adoption into human society as companion animals (‘pets’) may have altered the predator–prey dynamic, brought aspects of wild pig behaviour and reproduction under indirect human selection and control, and caused changes that differentiated human-associated pigs from their solely wild-living counterparts.

## 1. Introduction

The prevailing view on the beginnings of animal domestication is that the first taxon domesticated by humans was the wolf (*Canis lupus*), giving rise to domestic dogs (*C. familiaris*) [1,2,3,4,5,6,7,8]. Scholars and scientists have widely contended that the domestication of a now-extinct variant of this wild carnivore was initiated somewhere in Late Pleistocene Eurasia, including eastern Asia (e.g., China), by a non-sedentary population of anatomically modern human (AMH) hunter-gatherers, certainly by at least 15–16 thousand years (ka) ago [9], but possibly up to 40 ka ago according to some authorities [10]. The current thinking is that no other animals were domesticated by humans until after the advent of both plant food agriculture and the rise of settled village life (sedentism) around 11.5 ka ago in Southwest Asia [4]. Archaeologists infer that, at this point in time, some human populations in the region were transitioning from a foraging lifestyle to raising cultivated crops in permanent settlements. It was supposedly only at this critical juncture in world history that our species began to domesticate other animals, beginning with our ‘Big Four’ earliest domestic livestock species: pigs (*Sus scrofa*), goats (*Capra hircus*), sheep (*Ovis aries*), and cattle (*Bos taurus*) [4]. (*S. scrofa* was also independently domesticated during the Neolithic period in China [11]). Under this scenario, the domestication of *C. lupus* was an unprecedented event in the evolutionary history of our species; in the sense that it was initiated by small, mobile groups of Late Pleistocene foragers who had no pre-existing knowledge of cultivation or animal domestication (but cf. [5]). 

The Sulawesi warty pig (*Sus celebensis*) is perhaps the only other animal species for which there is a longstanding claim for a domestication event involving a pre-agricultural human population (reindeer and horse are two other possible examples [4,12], but it is beyond the scope of this paper to discuss them here). Also known as the Celebes wild boar, *S. celebensis* is a still-extant, free-roaming suid that is endemic to Sulawesi [13], the largest of the ‘Wallacean’ islands situated between the continental landmasses of mainland Southeast Asia (Sunda) and Australia–New Guinea (Sahul). In a landmark study on the taxonomy and phylogeny of the Suidae, the late Colin Groves (1942–2017) proposed [14] that humans may have independently domesticated *S. celebensis* at some stage prior to the late Holocene expansion of Austronesian-speaking agricultural populations and their Neolithic cultural package into Island Southeast Asia [15]. If so, *S. celebensis* is the only known species of suid other than the Eurasian wild boar (*S. scrofa*) to have been successfully domesticated by humans [14,16].

The Austronesian diaspora led to the initial human settlement of remote Oceania (in the guise of the succeeding Lapita culture) and reached as far west as Madagascar [15]. Current evidence suggests that the spread of Austronesian-speaking societies from outside of their Taiwan homeland commenced ~3 ka ago [17] and was driven (after a time lag) by the emergence of rice-based agriculture in southern China. This date comes from early pottery finds and is imprecise [17]; earlier chronologies for the Austronesian dispersal ranging from 5.5 to 4 ka ago have been proposed [15,17]. Whatever the precise timeframe, it has long been argued that domestic dogs (*C. familiaris*), pigs (*S. scrofa*), and chickens (*Gallus gallus*) accompanied Austronesian settlers on their migratory journeys through Island Southeast Asia and out into the western Pacific [15,18,19]. There are problems with this characterisation of the Austronesian incursion as signalling the wholesale import of the first farming culture to the region, which are discussed below. For example, in reality, the presence of the skeletal remains of domestic dogs, pigs, and chickens in the earliest Neolithic levels in the region is ‘extremely uneven’ ([20], p. 25). Nevertheless, based on the evidence available to him at the time, Groves [14] proposed that prior to the Neolithic local groups of seafaring hunter-gatherers had carried Sulawesi warty pigs with them on ocean voyages and released them on several islands; perhaps to stock faunalistically impoverished islands with a source of animal protein. This early human-mediated dispersal of an endemic pig was then over-printed by the introduction of the Austronesians’ domestic swine (*S. scrofa*), with this second wave of pig translocations replacing *S. celebensis* in all but the most outlying Indonesian islands [14]. 

It is difficult to evaluate Groves’s proposition because so little research has been conducted into the natural history of *S. celebensis*, and certainly not into the pattern of human relations with this species in the deep past (but see below). Despite this dearth of empirical evidence, however, the basic premise of Groves’s argument that *S. celebensis* was domesticated and widely translocated by pre-agricultural foragers is often referenced in general scholarly treatises on animal domestication and the history of suids in particular (e.g., [21,22]). It has also seen broad acceptance among authorities in the field (e.g., [13,20,23,24,25]), including key specialists in the taxonomy, ecology, and natural history of *S. celebensis*. As one group of scholars has commented, for instance:

The [Sulawesi warty pig] has also been introduced elsewhere in Indonesia, e.g., to the islands of Flores, Timor, Lendu, and Simeulue. The wild pigs on some of these islands are strongly modified and there is now little doubt that *S. celebensis* has been domesticated and transported to these areas as a domestic or feral form, probably during the early migrations of peoples.

([13], p. 185)

Groves’s argument was also given new impetus in the late 2000s when Larson and colleagues [24] published a *S. celebensis* mtDNA sequence extracted from a suid tooth that had been excavated from Liang Bua cave in Flores, and which, according to this team, dated to ~7 ka ago. While some archaeologists have viewed this claim with circumspection, especially the early date [19,20]—for which there is some justification (see Appendix A)—other authorities have accepted it at face value, seeing it as evidence for the human-mediated translocation of *S. celebensis* during the middle Holocene. This view is apparent in the most current discussions about this pig [13]. 

While Groves’s decades-old idea that *S. celebensis* was independently domesticated in the pre-Neolithic period is an accepted feature of the scientific literature, an in-depth critique of this proposition has not been published before. Hence, the aim of this paper is to evaluate this long-standing notion from a critical perspective. This will be achieved not by rigorously assessing the evidence Groves presented in support of his argument (i.e., claims for living populations of *S. celebensis* outside Sulawesi, including domesticated variants)—this is beyond the scope of this paper—but rather by examining a quandary that Groves did not attempt to resolve, perhaps sensibly, in his long-running commentary on this pig (for his most recent publications on the subject, see [26,27]). Specifically, is it realistic to suggest that pre-agricultural foragers could have entered into a relationship of domestication with a wild suid, thus achieving something that no other non-sedentary hunters and gatherers had achieved at any period in world history? Put another way, is Groves’s scenario so highly improbable that it can be dismissed out of hand; that is, without the need to find evidence to refute it? Or does it have support in what is presently known about early human–suid relations in Sulawesi and the wider region?

## 2. Groves’s View on the Early History of the Sulawesi Warty Pig

**Figure 1:**
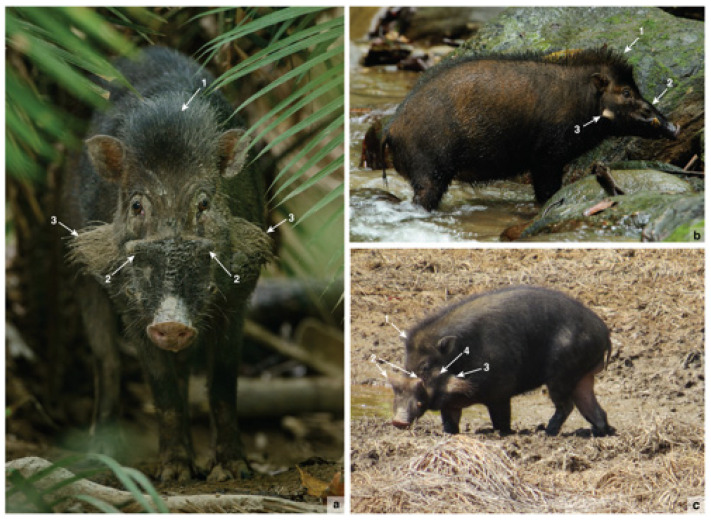
The Sulawesi warty pig (*Sus celebensis*). Characteristic morphological features include: 1 = head crest; 2 = preorbital facial warts; 3 = mandibular (gonial) warts and associated hair whorls; 4 = postorbital facial warts. The photographs were all taken in nature reserves on Sulawesi. The individual in (**c**) is an old male with pronounced postorbital facial warts. Image credits: (**a**,**b**), C.C. Lee; (**c**), A.H. Mustari.

*S. celebensis* is a small pig with distinctive facial warts (Figure 1). Among the most diminutive of the still-extant Southeast Asian suiforms [16], it is still found in the wild in relative abundance in various parts of Sulawesi today [13]. The idea developed by Groves [14,16] that *S. celebensis* was domesticated by pre-Neolithic peoples and then widely translocated in Indonesia involved inductively piecing together a narrative after the fact based on various observations rather than proposing and testing a formal hypothesis. His decades-old proposition is based on two key strands of evidence: the complex biogeographical distribution of *S. celebensis* populations, implying a pattern of human-mediated dispersal at some stage in the distant past, and the apparent presence of *S. celebensis* pigs living in the company of humans in a state of domestication.

**Figure 2:**
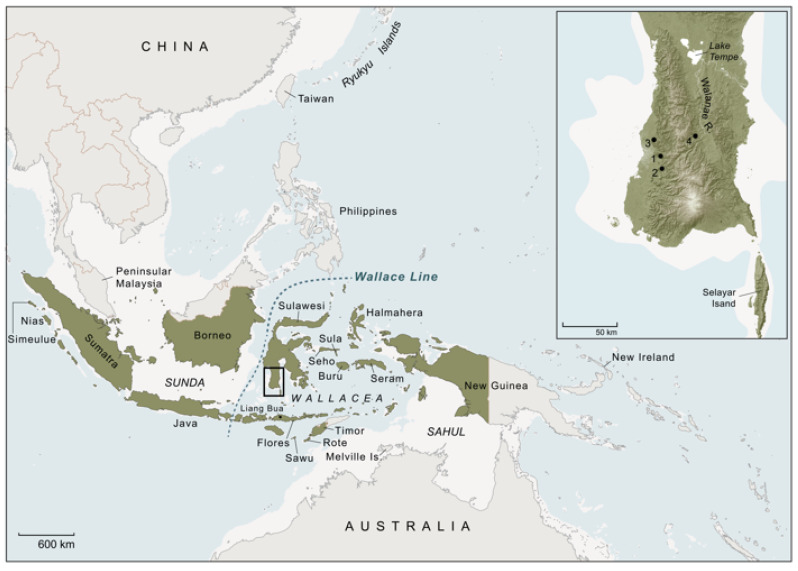
Island Southeast Asia and the wider region. Islands and provinces of the Republic of Indonesia are shaded green. White shading represents land areas exposed during Late Pleistocene low sea level stands. The Wallacean archipelago (Wallacea) is located east of the Wallace Line, the biogeographical boundary delineating the easternmost distribution of the floral and faunal worlds of the Southeast Asian continental shelf (Sunda), and west of the Australia–New Guinea landmass (Sahul). The present-day distribution of the Sulawesi warty pig (*S. celebensis*), based on current assessments or conjecture (data sources: [14,28,29]), includes Sulawesi, where this suid taxon is an ancient component of the insular mammalian fauna, as well as Flores, Sawu, Rote, and Timor, all of which are Wallacean islands where *S. celebensis* is held to have been introduced by humans in pre-Neolithic times. The species is also reputed to have been translocated to New Guinea, and apparently as far west as Simeulue Island (and Nias). Rote is the only island (including Sulawesi) where it is claimed that fully domesticated *S. celebensis* pigs have been observed in modern times [14]. Inset panel shows the southwestern peninsula of Sulawesi. The numbered dots are the locations of key archaeological sites mentioned in the text. Cave sites with dated Late Pleistocene rock art depictions of *S. celebensis* pigs: 1, Leang Tedongnge; 2, Leang Balangajia 1; 3, Leang Bulu’ Sipong 4; excavated Toalean site: 4, Leang Panninge. Sites associated with the local Holocene foraging culture (Toalean) are only found south of Lake Tempe. Base map courtesy of K. Newman.

With regard to the first form of evidence, based on his morphological analyses of suid skins and skulls in museum and natural history collections Groves [14] concluded that *S. celebensis* also occurs in the fauna of Simeulue, a small Indonesian island located off the west coast of Sumatra 2600 km to the west of Sulawesi, as well as in New Guinea 630 km to the east (Figure 2). He further contended that distinct populations of *S. celebensis* are found some 300 km to the south of Sulawesi in the Lesser Sunda islands (Flores, Rote, Sawu, Timor) on the southern margin of Wallacea, and possibly also in the Maluku Islands (Moluccas) of Halmahera, Buru, and Seram, although with lesser certainty in these latter cases (Figure 2) [14,16]. Based on what he described as this ‘zoogeographically implausible’ distribution, Groves ([16], p. 112) proposed that one or more groups of pre-Neolithic foragers must have used watercraft to transport *S. celebensis* pigs to other islands in Indonesia, either in the form of domesticates, feralised descendants of domestic livestock, or as wild animals kept in human captivity Groves ([16], p. 117).

It is important to note that the translocation of animals by watercraft does not necessarily equate to domestication per se. For example, early Neolithic peoples introduced the wild fallow deer (*Dama dama mesopotamica*) to Cyprus, supposedly to replenish the island with game for hunting, yet they never domesticated these cervids [30]. Much the same thing happened with wild deer (*Rusa timorensis*) in prehistoric Wallacea, although it is uncertain when the human-mediated dispersal of this taxon began [31]. It is also thought that foraging peoples translocated the northern common cuscus (*Phalanger orientalis*) from mainland New Guinea to New Ireland around 23.5–20 ka ago [32]. In the wider region, wild boar (*S. scrofa*) may have been carried by watercraft to the Ryukyu Islands of Japan [33,34], perhaps by 27–24 ka ago [35] (but for conflicting genetic data, see [36]). On the other hand, the human translocation of wild suids to Cyprus at the tail end of the terminal Pleistocene is seen as evidence for a human-imposed system of game management and ‘control in the wild’ ([28], p. 131), and thus of an increasingly close relationship between people and *S. scrofa* [37].

**Figure 3:**
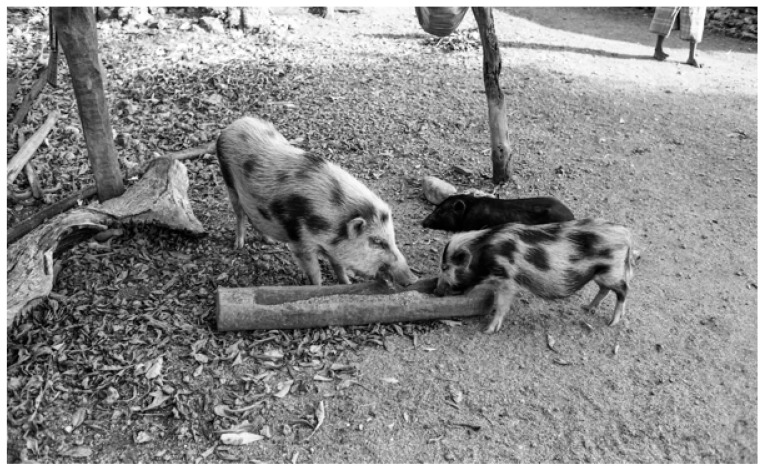
Village pigs on the eastern Indonesian island of Rote. Anthropologist James Fox took this photograph in or around 1972. These pigs are among a population identified by Colin Groves [14], based on Fox’s images, as domesticated variants of *S. celebensis*, based both on their status as village pigs that lived with humans and the presence of external morphological characteristics associated with domestication (e.g., piebald coats, visible in this image). Credit: J. Fox.

Groves’s [14,16] conjecture that *S. celebensis* had been independently domesticated in pre-Neolithic times was principally based on his observations of pigs that seemed to be cohabitating with farmers as fully domesticated swine on the eastern Indonesian island of Rote in the 1970s. His published descriptions of these particular animals appear to have been limited to his evaluation of anthropologist James Fox’s film footage and photographs of pigs owned by settled communities of lontar palm harvesters (Figure 3). Fox’s imagery showed small herds of these pigs being housed in villages and fed at troughs. (Fox describes these suids as being kept under similar conditions as ‘pets’, noting that they insisted on keeping in close contact with villagers for scraps of food [J. Fox, pers. comm., 2017]). According to Groves ([14], p. 63), the pigs seen in these Rote villages displayed a mix of morphological attributes one might expect to find in *S. celebensis* pigs that manifested traits associated with domestication, such as facial warts (a diagnostic trait of *S. celebensis*) and piebald coats (a character of domestic *S. scrofa* swine). In the wider context of his model, Groves’s assessment of the Rote pigs implied that domesticated variants of *S. celebensis* had been more widespread in the region prior to the Austronesian expansion and that living representatives of this ancient line of domesticates could now be found only as a remnant group on this one outlying Wallacean island.

Current evidence generally supports Groves’s original claim that *S. celebensis* is (or was) found well outside its natural range. For example, empirical support for the presence of *S. celebensis* pigs far to the west of Sulawesi, in Simeulue Island, is provided by recent genetic admixture analyses, which reveal gene flow from *S. celebensis* into local *S. scrofa* populations on Sumatra and in mainland Southeast Asia [38]. Groves’s claim [14] that New Guinea pigs are hybrids of *S. scrofa* and *S. celebensis* has been contested [24]. However, Ishiguro and colleagues ([29], p. 9) identified a New Guinea *S. scrofa* sample with a *S. celebensis* mtDNA haplotype, suggesting ‘The ancestor of this mtDNA haplotype might have also been introduced to New Guinea from Sulawesi by ancient peoples’. Field observations from central Flores are also consistent with the presence of a free-ranging population of *S. celebensis* in the extant island fauna [39].

With regard to Groves’s idea that *S. celebensis* was independently domesticated prior to the migration of Austronesian-speaking Neolithic farming societies into the region, as yet, there have been no attempts to test this specific proposition using zooarchaeological evidence. Indeed, on the basis of the available records, it may not yet be feasible to do so, owing to the scanty record of well-described pig remains excavated from reliably dated archaeological contexts in the key islands of interest. While there is sufficient data to suggest that prehistoric Indonesians translocated *S. celebensis* to other islands–although precisely when they began doing this is unclear—clear support for its pre-Neolithic domestication is lacking. As noted, Groves [14] argued that living members of this species exist (or did until recently) in a domestic partnership with villagers on the island of Rote in eastern Indonesia. Investigating this issue is beyond the scope of the present paper (but see [40,41]). In any case, it would not necessarily address the problem, given that modern domestic populations of *S. celebensis* (if they exist) could have been domesticated by village farmers in recent times.

## 3. Current Theories on the Domestication of *S. scrofa*

Defining the early human–animal relations that provided the impetus or laid the groundwork for the domestication of the wild-living ancestors of modern domestic species is invariably conjectural, requiring a considerable amount of scholarly deduction and guesswork [42,43,44]. In the case of the wolf, two competing hypotheses have been proposed to explain how a non-sedentary population of Late Pleistocene foragers could have domesticated this large predatory canid [45,46,47,48]. The first hypothesis is that the wolf ‘self-domesticated’ by establishing a commensal relationship with mobile groups of AMH hunter-gatherers [4,7,47,49,50,51,52,53,54,55,56,57,58]. The second hypothesis, now increasingly seen by scholars as being more tenable than the first (but cf. [59], p. xi), is known variously as the human-initiated, cross-species adoption, pup-raising, or pet-keeping model [45,46,47,48,60,61,62]. The basic premise is that Late Pleistocene foragers reared and socialised wild-caught wolf pups as companions (‘pets’), causing genetic changes in the captive populations that gradually differentiated them from purely wild, free-ranging wolves [45,46,47,48].

In contrast, the current thinking is that the ruminant livestock (goats, sheep, cattle) followed a ‘predator’ pathway to domestication, whereby increasing human population levels in early Neolithic settlements led to over-hunting of these wild prey and the development of game management strategies (e.g., selective culling of males) in response [42,43,44]. These interventions in the reproductive processes of wild-living ungulates supposedly then intensified to the point that humans were actively managing and controlling herds in the wild, an early step in the eventual transition to selective breeding [42,43,44]. However, the domestication of *S. scrofa*, an omnivore with a flexible diet that differs markedly from that of the ruminant livestock, is seen as involving a more complicated process [11,37]. Price [37] provides the most detailed and up-to-date version of the standard account of the beginnings of the domestication process in pigs, which, he argues, involved the following sequence of events:

1. At the beginning of the Holocene, hunter-gatherers in Southwest Asia (Pre-Pottery Neolithic A culture [PPNA, 9700–8500 BC]) began settling down in village communities sustained by incipient forms of plant food cultivation;
2. PPNA people used early domestic dogs to hunt wild Eurasian boar, employing packs of trained canines to track down wild boar by smell and keep them at bay for hunters to dispatch safely at a distance; prior to dog-assisted hunting, it is surmised, wild boar were avoided as prey by western Eurasian foragers because these animals were too elusive and dangerous to hunt, and hence the model assumes that early AMH foragers in Upper Palaeolithic Europe and during most of the Epipalaeolithic period in the Near East ate no, or very little, pork;
3. Human population growth in year-round PPNA settlements intensified the pressure on local wild boar numbers, leading hunters to devise game management strategies to maintain this dwindling food resource, such as selective culling of males and translocation of wild boar from the mainland to isolated islands (i.e., Cyprus [63,64,65]);
4. Wild boar attracted by cultivated crops and agricultural refuse become established as a commensal pest species around village settlements;
5. Commensal wild boar began interbreeding with human-managed wild boar;
6. Hunters increasingly targeted farrowing wild sows—which are vulnerable to predation because they customarily leave their social groups, or sounders, to give birth—in order to obtain wild piglets, which were hand-raised back at the settlement for slaughter and consumption at a later time;
7. The human–pig bond was maintained by provisioning captive adult pigs with fodder derived from crop-raising;
8. Once PPNA people began to control wild boar herds and actively interfere in the animals’ daily lives and reproductive processes, they inadvertently selected for the mutations associated with domestication traits (e.g., behavioural tameness);
9. Domestication occurred when densely populated PPNA communities were raising and breeding managed herds of wild boar inside permanent village settlements. By the onset of the Pre-Pottery Neolithic B period (PPNB, 8500–7000 BC), human management and control of captive wild boar had intensified to the point that the gene flow from the free-ranging population was disrupted for long enough for some domestication traits to become fixed.

Notably, the model for the domestication of *S. scrofa* in Southwest Asia involves a complex mix of game management and commensalism, with settled communities of incipient farmers and pig hunters slowly changing the nature of their relationship with the local wild boar populations that lived in the vicinity of their settlements. The independent domestication of *S. scrofa* is thought to have occurred in the river valleys of China by around 6600–5000 cal. BC [11]. Less is known about the earliest stages of pig domestication in this region of eastern Asia. Based on current evidence, the process appears to have started at around stage 4 of the Southwest Asia model and was broadly similar, although human control over pig breeding possibly occurred earlier in China [11]. 

## 4. Archaeological Evidence for Early Human Interactions with *S. celebensis*

What is the present state of knowledge about the nature of the relationship between pre-Neolithic foragers and wild-living *S. celebensis*? Current archaeological evidence suggests AMHs have been established in Sahul for at least 50 ka [66] and possibly up to 65 ka [67]. The initial peopling of Sahul would have required the first major ocean crossing undertaken by our species; however, migrating east of Sunda through the Wallacean islands also necessitated multiple maritime voyages and associated colonising events [68], including the colonisation of Sulawesi by 45.5 ka ago (see below). 

The first AMHs to travel to Sulawesi from the adjacent mainland with its classic Asian fauna would have encountered a highly exotic animal world [69]. The biogeographical isolation of this large landmass and its proximity to the Asian mainland have given rise to an exceptionally high rate of species endemism, especially in mammals [70,71,72]. Some 98% of non-volant mammal species are endemic [73]. The largest of the still-extant non-flying land mammals are a dwarf bovid (anoa, *Bubalus* sp.) [74,75,76] and two Suidae genera: *Babyrousa* (babirusa) [77] and *Sus* (*S. celebensis*) [13,14,72,78]. The remaining insular taxa consist of medium- to smaller-sized animals: tarsiers, macaques, a civet, two cuscuses, and an array of rats, shrews, and squirrels [71,72]. Sulawesi’s mammalian apex predator is a 4–6 kg civet (*Macrogalidia musschenbroekii*) [79,80].

**Figure 4:**
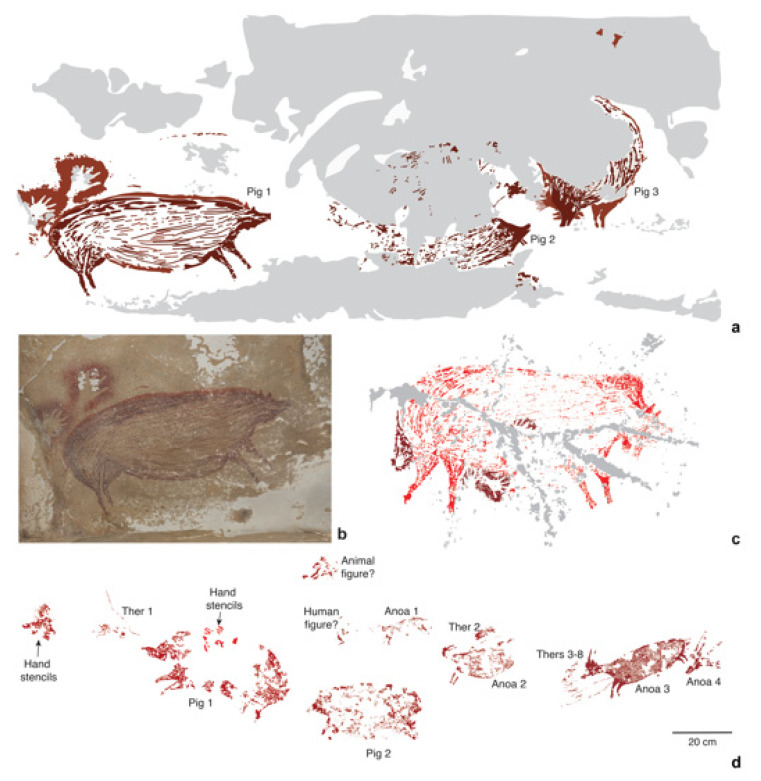
Dated Pleistocene rock art images of *S. celebensis* from caves in Sulawesi. (**a**), Digital tracing of a rock art panel from Leang Tedongnge, depicting three *S. celebensis* individuals (Pigs 1–3); (**b**), photograph of Pig 1, which has been dated using the U-series method to at least 45.5 ka [83]. Dimensions: Pig 1 (136 × 54 cm), Pig 2 (125 × 53 cm), Pig 3 (138 × 71 cm). (**c**), Digital tracing of a painting of a *S. celebensis* pig (187 cm in length and 110 cm in height) at Leang Balangajia 1, which yielded a minimum U-series age of 32 ka [83]. (**d**), Digital tracing of a rock art panel portraying a hunting ‘scene’ at Leang Bulu’ Sipong 4. U-series dating shows that this narrative composition was painted at least 43.9 ka ago [82]. It depicts small therianthrope-like figures (Thers 1–8) using spears or ropes to hunt *S. celebensis* pigs (Pigs 1–2) and dwarf bovids (Anoas 1–4) [82]. Grey shaded areas in (**a**,**c**) represent exfoliated portions of rock art panels. Credits: (**a**,**c**,**d**): A.A. Oktaviana; (**b**), M. Aubert.

It has recently been established that Sulawesi is host to some of the oldest dated rock art in the world [81,82] and possibly the earliest known artistic representations of animals found anywhere [83]. The oldest dated rock art on the island occurs in the limestone ‘tower’ karst district of Maros-Pangkep in South Sulawesi and features early figurative depictions of *S. celebensis* pigs [41,83]. Thus far, the earliest motif identified is a painting of a male suid from the limestone cave of Leang Tedongnge (Figure 4a,b), which has a minimum Uranium-series age of 45.5 ka [83]. This is currently the earliest proxy evidence for our species on the island. The dated suid motif is identified as *S. celebensis* based on the figurative representations of facial warts and other species-specific morphological characters that were captured by the artist(s) with considerable anatomical fidelity [41,83] (Figure 4a,b). This artwork is part of a larger narrative composition or ‘scene’ that features two other naturalistic representations of *S. celebensis* [41,83]. A large and impressive figurative painting of a Sulawesi warty pig was also dated to at least 32 ka ago at Leang Balangajia 1 [83] (Figure 4c).

Notably, at Leang Bulu’ Sipong 4, another cave site in this karst area, a composed rock art scene painted at least 43.9 ka ago (Figure 4d) seemingly depicts therianthropic (part-human, part-animal) figures hunting *S. celebensis* pigs and anoas [82]. The apparent portrayal of therianthropes in this artwork could imply an early conception among Sulawesi foragers that humans were connected to the non-human animal world on a symbolic (and possibly spiritual) level, and perhaps to certain species in particular. Concerning the latter, over 87% of animal motifs in the Pleistocene rock art style phase seem to represent *S. celebensis* pigs, offering hints at the importance of this species in the cultural world of the early AMH communities on the island [41]. 

In addition, excavated archaeological findings show that pre-Austronesian foragers in the Maros-Pangkep karsts exploited *S. celebensis* as a prey species for tens of thousands of years [69,84]. Hunting methods used by Late Pleistocene people to capture *S. celebensis* pigs are unknown. The above-noted cave painting at Leang Bulu’ Sipong 4, dating to at least 43.9 ka ago, seems to depict human-like figures hunting adult *S. celebensis* pigs and anoas with long thin objects that can be interpreted as representing spears [82]. Alternatively, the lines portrayed in this pictorial narrative can be construed as long ropes, which could imply that wild-living adult *S. celebensis* pigs were sometimes captured alive [82]. It is worth noting that present-day foragers in the region occasionally capture small wild juvenile suids and bring them back to their camps with the intention of using the live animals to ‘blood’ young hunting dogs (e.g., the Penan of Borneo; [85], p. 249).

A particularly heavy reliance on *S. celebensis* is evident among the ‘Toalean’ hunter-gatherers of the middle to late Holocene period (~8 to 1.5 ka ago) in South Sulawesi [86,87,88]. These Holocene-era foragers inhabited the same caves and shelters containing the much earlier rock art images of *S. celebensis* [41,83], but the relationship of the Toalean culture to the Late Pleistocene artistic culture remains uncertain. In the Maros-Pangkep karsts, the Toalean ‘heartland’, faunal collections are dominated by *S. celebensis* (up to 65% of identifiable elements at some sites), while it is around 50% on the south coast of the peninsula [84]. This zooarchaeological evidence testifies to what some specialists have described, speculatively, as a peculiarly close relationship between *S. celebensis* and the Toaleans: ‘While we lack morphological evidence to suggest that the Holocene Toalean hunter-gatherers in this locality had domesticated [*S. celebensis*], there must certainly have been a commensal or even a mutualistic relationship’ ([84], p. 177). Elsewhere, Bulbeck writes: ‘*Sus celebensis* so dominates the pre-ceramic Holocene faunal remains from the Maros sites in South Sulawesi that it was minimally a commensal and conceivably a domesticate’ ([89], p. 34). It should be noted that Bulbeck’s perspective on this matter, as evident from these comments, appears not to have been based on any direct evidence for domestication discerned in the faunal assemblages under study.

No rigorous studies have been conducted on *S. celebensis* remains from archaeological assemblages of any age or association to specifically determine if these Sulawesi warty pigs exhibit diagnostic traits of domestication visible in skeletal materials (e.g., reduced cranial capacity). However, based on the faunal assemblage excavated from Toalean deposits at Leang Panninge (Maros), Saiful and Aggraegani [90] contend that there was a rapid increase in the number of juvenile and immature suids in the late Holocene, coupled with a rise in rates of linear enamel hypoplasia (LEH) in suid teeth. This apparent evidence for changing mortality patterns and a rise in an indicator of developmental stress (LEH) is noteworthy but requires further validation. The authors suggest it could be explained by the emergence of pig domestication, perhaps initiated by Toalean hunter-gatherers who observed the domestic *S. scrofa* of the Austronesian-speaking farming societies that had begun to settle on the island. In their study, however, Saiful and Aggraegani [90] were unable to reliably distinguish between *S. celebensis* and babirusa molars selected for analysis. Some analysed samples could possibly be domestic *S. scrofa*. Hypoplasias also have a wide range of explanations, and LEH defects are not necessarily causally linked to domestication [11].

There is no evidence that any of the pre-Austronesian peoples of Sulawesi or the wider region were sedentary and/or engaged in food production analogous with that of the PPNA/PPNB peoples of Southwest Asia, where the beginning of pig domestication is inextricably linked to the ‘Neolithisation’ of human society [11,37]. It is possible that sedentary farming was present in the region prior to the Austronesian expansion, and evidence for it has not yet been identified [91]. On the other hand, the notion of a strict dichotomy between the foraging economies of pre-Neolithic Island Southeast Asia and the settled, agrarian lifestyle of incoming Austronesian populations is questionable [89]. In fact, the Neolithic in this region generally does not conform to the classic image of the development and, or the arrival of a single culture that included agriculture, sedentism, animal domestication, pottery production, and lithic technology innovations [20,92]. 

To begin with, it is evident that people in the highlands of New Guinea were using wetland agriculture to cultivate indigenous plant foods (e.g., banana [*Musa* spp.]) in the early to middle Holocene (~8–4 ka ago) without having any apparent knowledge of domesticated animals (but see below) and pottery [93]. Furthermore, genetic analyses suggest that in Indonesia the cultivation and/or management of indica rice (a sub-variety of *Oryza sativa*) might actually pre-date the arrival of the Austronesians [94]. There are also hints that some indigenous groups in Island Southeast Asia (especially those inhabiting tropical forests) were already cultivating or managing root and tree crops before Austronesian-speaking populations expanded into the region [20]. Indeed, it is conjectured that ‘hunter-gatherers’ in lowland New Guinea could have employed systems of arboriculture that facilitated the emergence of large semi-sedentary societies, but that entailed methods of wild tree-exploitation (e.g., sago pith extraction using non-lithic technology) that would be almost impossible to detect in the archaeological record [91,92,95,96].

What is more, actual evidence that incoming groups of Austronesian speakers were sedentary cereal cultivators is rather sparse [20,89,91]. In fact, early Neolithic assemblages in much of the region can be interpreted to suggest that the Austronesian world was characterised more by a maritime-oriented lifestyle focused on nomadic fishing/foraging and extensive sea-trading than farming and living inland in permanently settled villages [89,91]. Indeed, the food crops that the Austronesians exploited (mostly tree and tuber cultigens) may have been co-opted from indigenous groups [89,91]. 

## 5. A New Model for Human–Suid Interactions in Pre-Neolithic Sulawesi

As noted earlier, a central proposition of the standard model of *S. scrofa* domestication is that a domestic partnership emerged relatively rapidly (in terms of archaeological timescales) between Southwest Asian foragers and an animal that their AMH predecessors had hitherto ignored or ranked very lowly as a prey species, and thus had limited or no close interactions with in the past [37]. This differs markedly from the situation in Sulawesi. Here, it is evident that foragers had intensively hunted a wild suid taxon within an insular island environment for tens of thousands of years—from the Late Pleistocene through to the pre-Neolithic Toalean cultural period—and clearly without the use of domestic dogs; indeed, presumably, these people did not even have direct knowledge of the existence of canids. The early peoples of Sulawesi thus had ample time to interact closely with this wild-living species of suid and accumulate detailed knowledge of its ecology, physical and behavioural characteristics and reproductive processes, and develop a cultural tradition of myths and stories related to this pig (as inferred from the apparent preoccupation with *S. celebensis* in the surviving record of the island’s earliest artistic culture). Of course, the presence of a long-term predator–prey relationship does not lead automatically to domestication; for instance, Neanderthals intensively hunted wild equids without ever domesticating the horse, as did AMHs in Upper Palaeolithic western Europe. The crucial factors in the standard model for the Neolithic domestication of *S. scrofa* were the rise of settled village life and plant food production (agriculture): humans living in permanent settlements sustained by crop-raising were able to maintain the human–pig bond in the long term by keeping pigs attached to a village community (though still allowing them to forage independently) and provisioning them with fodder derived from cultivated plant food [37]. In this way, early farmers were able to maintain a breeding population of pigs that was isolated from wild boar populations for long enough to initiate the genetic changes responsible for the emergence of domestication traits [37]. However, what if human populations in pre-Neolithic Sulawesi were able to independently converge on essentially the same outcome following a different trajectory? 

What if, over the long history of their association with wild *S. celebensis* pigs as a hunted game species, people who did not live in settled villages and had no knowledge of agriculture as conventionally defined interacted closely enough with Sulawesi warty pigs to cause these human-associated populations to change? This might not equate to pig domestication sensu stricto, but it could be an important step in that direction. It at least would represent a shift in forager–wild suid relations that has not yet been considered in the Southwest Asia-centred model of pig domestication. How could such a human–suid relationship have developed, and what would it have looked like? 

Here, it is proposed that it would not have been based on the control and exploitation of pigs as livestock for meat production, but rather on the cross-species adoption of wild piglets. As noted, the classic model of pig domestication in Southwest Asia involves incipient farmers socialising wild-caught piglets from an early age to form emotional attachments with individual human caregivers [37], thus intervening in the natural filial imprinting process—as ubiquitously observed in Melanesian pig-rearing cultures [11,97,98,99,100]. In fact, the first Neolithic domestic pigs reportedly can be traced back to these hand-reared, human-socialised, wild-born juvenile boars [37]. Moreover, as has already been mentioned, various authorities maintain that foragers in Late Pleistocene Eurasia formed a bond of interspecies companionship with the wild wolf progenitors of dogs, capturing pre-weaned pups and rearing them as pets [45,46,47,48]. 

It has also already been speculated that the relationship that gave rise to the first domesticated Eurasian wild boars (*S. scrofa*) could have been based on humans keeping pigs as dog-like pets. For example, Sauer ([61], p. 31) suggested that wolves and *S. scrofa* pigs were domesticated in similar ways: by humans taking pre-weaned piglets and puppies from the wild and raising them as household members, a process in which lactating women suckled young adopted animals (cross-species wet nursing) was pivotal. Simoons and Baldwin ([101], p. 436) also surmised that the origins of both dog and pig domestication might have been rooted in ‘a kind of “institutionalized” petkeeping, in which both the capture and the nursing of infant wild animals [were] integral parts of the system’. More recently, Serpell [62] conjectured that the same pet-keeping practices (cross-species adoption) that potentially transformed wolves into dogs [45,46,47,48] may account for the origin of other domesticated animals, including wild suids.

The argument comprises the following elements:

### 5.1. Pet-Keeping Is a Common Feature of Known Foraging Societies

Rearing wild animals as pets is a very widespread human behaviour, including among historically known foragers. The evidence for this is extensively documented and well-known. Briefly, the practice of adopting and/or caring for non-human animals is a pervasive phenomenon among historically known human societies [62,102,103,104,105]; although it is apparently not universal [106], and definitions of what constitutes a pet vary on a cross-cultural basis [107]. A pet is here defined as any animal ‘kept for seemingly non-subsistence reasons (i.e., not kept just for meat or other food products such as milk) but also for some apparent degree of companionship’ ([108], p. 19; see also [104]). In modern Western societies, it is common for pets to have a childlike role and to be deeply integrated into human families and social groups [105,106]. Similarly close attachments between humans and companion animals have been documented among various small-scale non-industrialised societies [104,105]. This includes ethnographically known foraging communities, where the ‘the owner–pet relationship… is typically understood as analogous to that between parent and child’ ([106], p. 298). 

An oft-noted example of foragers keeping wild-born animals as pets comes from Australia. Available accounts suggest that Aboriginal men, women, and children were besotted with wild-caught dingo (*C. dingo*) pups, intensely nurturing the young canids and forming deep emotional bonds [109,110,111] that may have continued even after the mature animals returned to the wild to breed [112]. This close human–canine relationship seems to have flourished in even the harshest parts of the arid interior where food resources were exceptionally scarce, and despite the widely held view that these human-associated canids conferred no obvious economic benefits, or certainly were not consistently useful as hunting ‘dogs’ ([113]; but cf. [114]). The main role of socialised ‘camp dingoes’ seems to have been as childlike companions (pets) [112]. Similarly, Aboriginal foraging peoples in contact-era Australia cohabitated with a wide array of wild animal species other than dingoes, including captive birds, reptiles, and marsupials; these animals were all kept in Indigenous communities as pets, although they tended to be more transient companions [115]. To take one example, the Tiwi people of Melville Island refrained from killing the helpless young of possums they caught in the bush, as they felt ‘“too sorry”’ for them ([116], p. 34). Instead, baby possums were brought back home for adoption by small children, who delighted in playing and interacting with them. Although the mortality rate was high, ‘Such an animal might be old enough to survive the night, “sleeping in the camp,” in which case it would not be killed and eaten, for it would now be considered a member of the camp’ ([116], p. 35).

Pet-keeping, according to some authorities, is difficult to explain from an evolutionary perspective, as expending time, energy, and resources on keeping animals exclusively for companionship seems to confer no obvious benefits in terms of human survival and genetic fitness [106]. Scholars have proposed a number of reasons for the evolutionary origin of pet-keeping, ranging from it serving as an honest signal of a prospective mate’s capacity to rear and nurture human offspring, to our tendency to project human mental states onto non-human species (anthropomorphism) [106,107]. It is also conjectured that the human propensity for cross-species adoption is ‘a maladaptive consequence of misfiring of evolved parental urges’; in other words, a byproduct of hypernurturing behaviour ([107], p. 299). This might account for why so many pet-keeping traditions seem to be based on the intense human attraction to juvenile animals and adults bearing infantile characteristics (paedomorphic traits) [106].

There are grounds to suggest that keeping animals as pets has deep roots in the evolutionary history of our species (and genus [117,118]). Indeed, some authorities argue that pet-keeping of some kind is likely to have been widely practiced among Late Pleistocene AMH groups, although when, where, and how the practice emerged remain unknown and poorly investigated [21,46,62,105,106,119,120]. 

Notwithstanding this view on the possible antiquity of pet-keeping, in modern Western scholarship there is also a long-standing preconception (e.g., [121]) that the beginnings of animal domestication could not have been grounded in something as seemingly inconsequential as the human desire for interspecies companionship [107]. It is a commonly held assumption, rather, that there had to have been an explicitly economic motive behind the earliest efforts made by our species to bring non-human animal species into the human domain. In the case of pre-agricultural foragers, the onus has been on scholars to explain why hunting and gathering societies would have kept wild animals as companions, rather than simply eaten them, ‘as if the only sensible reason for keeping an animal is in order, ultimately, to devour it’ ([104], p. 166). This attitude has roots in the 19th century when pet-keeping was characteristically viewed as an extravagance of the wealthy classes and social elites or anomalous behaviour displayed by emotionally inferior members of society (e.g., women and children) [104]. Scholars of this period regarded foraging as a precarious and arduous lifestyle and consequently assumed that perpetually food-stressed foragers had neither the time nor inclination to keep animals as pets [104]. Anthropological science had, however, generally dispensed with these notions by the 1960s, showing that foragers are, or were, from an economic perspective, comparatively ‘affluent’. That is, it was recognised that groups that lived by hunting and gathering had ample time and resources to partake in activities formerly held to be the province of societies that had advanced further up the evolutionary ladder, among them the ‘unnecessary luxury’ of keeping wild animals as social companions ([104], p. 166).

Certainly in the case of pigs, the notion of foragers keeping suids just for companionship is, for the most part, incompatible with the present anthropological mindset in which pigs equal food: pigs are prey for hunters or farmyard animals; they are livestock, not our social companions [122]. However, this is a view that would probably not be shared by the many traditional cultures of eastern Asia and the Pacific, where pigs are deeply integrated into human communities and often fulfill a complex role as a source of protein *and* companionship [122]. (Similarly, for most Western peoples, dogs equal pets, whereas domestic canines are consumed as livestock in parts of the Asia-Pacific and also kept as pet-like companions; [123]). Recent cases illustrate the legal difficulties people in Australia and the US have faced in keeping a pig as an ‘emotional support animal’ (i.e., a pet), especially in urban areas [124,125], owing to the commonly held perception that these animals are illegally housed livestock rather than legitimate social companions. 

### 5.2. Pigs Can Be Good Pets

In fact, pigs possess a multitude of traits that facilitate their popularity as dog-like pets on a cross-cultural basis [126,127,128,129,130]. Much like dogs, domestic pigs are highly social, group-living animals that display high-level cognitive capacities and complex psychology, including persistent individual differences in behavioural and emotional traits (‘personality’) [127,129]. Domestic pigs and dogs also share similarities in their ability to form close social relationships with humans. Similar to dogs, pigs exhibit traits that enable complex patterns of socio-communicative interactions with people, including discrimination between familiar and unfamiliar humans, sensitivity to human social cues and attention states, and human proximity-seeking behaviour [126,127,128,129,130]. Given that selective breeding has focused mostly on livestock production traits (e.g., growth and reproduction), the ability of domestic pigs to form close relationships with humans is assumed to reflect an adaptation or continuity from the cognitive capacities and social behaviour of their wild-living forebears [127]. These particular observations pertain to *S. scrofa*; however, there is no obvious reason to suspect that similar traits are (or would have been) lacking in other species of wild *Sus*, including ancient populations of *S. celebensis* in Sulawesi and elsewhere within the putatively wide distribution of this species. 

In a key study, Gray and Young [108] analysed human–pet dynamics across a wide array of societies (N = 60), including hunter-gatherers and other small-scale groups (horticulturalists, pastoralists, farmers, and other types). These researchers found that while dogs, cats, and birds are by far the most popular pets cross-culturally, being present in 88.3%, 53.3%, and 28.3% of the 60 societies analysed, respectively, pigs fall into the next most popular category [108]. Pigs that fit the definition of pets are present in one in ten societies [108]. Moreover, in small-scale societies pigs are as equally appealing as pets as monkeys and other nonhuman primates with well-recognised social cognitive abilities. Pigs are also more widely kept as pets than are reptiles [108]. Many traditional societies that keep pigs as pets also hunt and kill them for food [62]. However, there are typically strong social and moral taboos in place against eating prey animals that had lived with humans as pets; a practice commonly regarded by these societies as disturbing and repulsive—in fact, consuming the flesh of a pet is explicitly likened to cannibalism [106].

Foragers and other traditional societies in Island Southeast Asia and adjacent regions often keep (or kept) wild pigs as pets. In Indonesia and neighbouring regions, it is certainly possible to find accounts of humans developing close relationships and strong emotional bonds with wild-living pigs [101]. For example, Evans ([131], p. 64) refers to accounts of Semang foraging women in peninsular Malaysia breast-feeding wild piglets, which they reared as pets. 

Furthermore, it is well documented that Kubo women in lowland New Guinea capture feralised (‘wild’) *S. scrofa* piglets and intervene in the filial imprinting process, isolating them from other wild-living piglets and nurturing and breastfeeding them for the first 3–4 months, forming a strong emotional attachment that lasts until the pig is slaughtered [132]. In fact, throughout much of New Guinea (and the wider Melanesian region), domestic pig husbandry and management are heavily reliant upon these close, individualised relationships between wild-caught piglets and their human caregivers [11,97,98,99,100,133]. Domestic pigs, although an economic commodity, are treated more like cherished, childlike companions than ordinary livestock [133]. Consider anthropologist Margaret Mead’s comment in a 1932 letter from the field in northeastern New Guinea: ‘Pigs are so petted and cosseted that they assume all the characteristics of dogs—hang their heads under rebuke, snuggle up to regain favor, and so on…’ ([134], p. 111).

**Figure 5:**
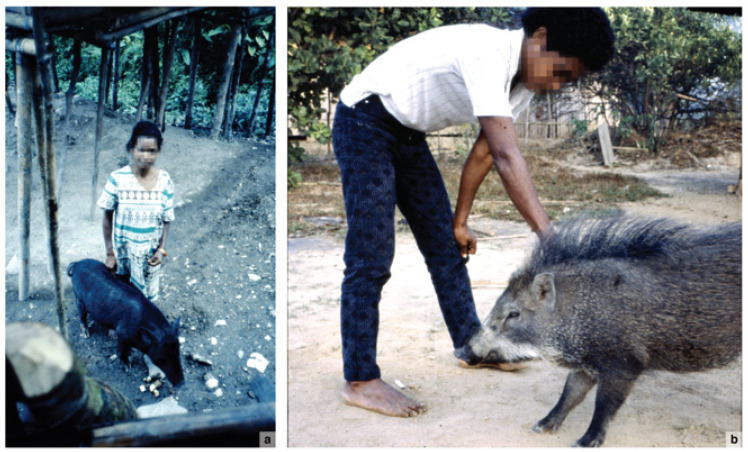
Wild pig companion animals on Seram, Indonesia. (**a**), Pepina with one of the wild pigs she befriended, a member of the family group that lived in the forest near her isolated home; (**b**), wild *S. scrofa* pig reared as a pet from an early age in Melinani village. Credit: A.A. Macdonald.

Another noteworthy example comes from the British zoologist and authority on the Indonesian Suidae, Alastair Macdonald, who reported a case in which an elderly Hindu-animist woman on the island of Seram (Maluku) formed a special rapport with a family group of wild pigs [135]. Pepina lived a solitary existence in a remote area at the foot of a mountain slope, where she regularly interacted with the wild pigs (presumably *S. scrofa*) that inhabited the forest surrounding her tiny house. The pigs routinely visited Pepina and fed on whatever scraps and refuse had fallen from the dwelling, which was positioned on stilts about 2 m above the ground (Figure 5a). Pepina named the pigs and knew them all intimately, including their individual vocalisations. Despite the fearsome local reputation of these animals, Pepina and the pigs co-existed harmoniously: ‘Pepina knew “her pigs” and they knew her. She was free to walk among them’ (A. Macdonald, pers. comm., 2021). Pepina even helped to keep these wild *Sus* pigs safe from predators, on one occasion rescuing her favourite sow, Sahli, from being killed by a python. After hearing Sahli squealing in the distance, Pepina ‘rushed through the forest to the aid of the animal. The snake had wrapped itself around the body of the pig and was compression-asphyxiating it. Pepina killed the snake [with a machete] and pulled its body off the sow ([136], p. 1). 

Macdonald is uncertain whether Pepina ever adopted any of the wild piglets from this forest-dwelling family group (A. Macdonald, pers. comm. 2021). Elsewhere in Seram, however, he observed the widespread practice of villagers capturing wild *Sus* piglets and raising them as household pets [135] (Figure 5b).

Although these accounts all involve human relations with *S. scrofa*, there is evidence that this potential for human–suid companionship was not particular to this taxon. In West Java, for example, it is reported that local hunters sometimes capture living Javan warty piglets (*S. verrucosus*) and bring them back to their village [137]. Similarly, Bornean bearded pigs (*S. barbatus*) are killed in large numbers by hunters during this suid’s seasonal mass migrations, especially when crossing rivers, and ‘Occasionally juvenile pigs are reared if they are captured alive’ ([138], p. 14). Elsewhere in Borneo, the Punan capture baby bearded pigs and feed and care for them until they become adults, when they are slaughtered and eaten (P. Piper, pers. comm., 2021). Penan foragers are also known to capture living *S. barbatus* piglets in the forest and bring them home for young children to play with as pets; in some cases, the young suids are deployed as target practice for small boys learning to use spears and blowpipes ([85], pp. 195, 260). Urquhart ([139], pp. 81–82) observed that the Penan ‘get very fond of young animals such as monkeys and wild [*S. barbatus*] piglets, even to the extent occasionally of women allowing these creatures to suckle them’. 

In Sulawesi, there seem to be no accounts of people keeping wild *S. celebensis* pigs as pets, but they are sometimes taken alive. For example, Blouch ([140], p. 7) observed a live *S. celebensis* sow (~30 kg) for sale at a market in Minahasa (northeastern Sulawesi). The vendor told him that the previous day he had sold eight live piglets of this species [140]. Babirusa also seems to have been reared as pets in the recent past. Indigenous peoples attest to this formerly widespread practice in Buru and the Sula Islands (Maluku) and closer to Sulawesi in Sehu Island (Seho). In one case, an elderly woman recalled two babirusa she had owned in her youth, including one that ‘became a longterm household pet and followed her around everywhere’ ([141], p. 28). Elderly residents of one Buru village also told of a babirusa that had formerly been raised as a pet within the community: ‘The villagers kept it in a cave and it wandered freely around the village like a dog’ ([141], p. 26; see also [142], p. 18 and [143], p. 22 for accounts of early European colonists in the region keeping babirusa as pets).

### 5.3. Pre-Neolithic Foragers on Sulawesi Probably Kept Young S. celebensis Pigs as Pets

It is contended, based on the evidence presented for pet-keeping practices among modern-day foragers and our understanding of human–animal companionship and cross-species adoption of young animals more generally [104,106,107], that early AMH hunter-gatherers on Sulawesi would have regarded juvenile members of *S. celebensis* as aesthetically appealing, and that these peoples had the capacity to form intense emotional attachments to them. It therefore seems possible that at least some pre-Neolithic foragers in the island would have kept wild juvenile pigs (and other animals) as pets. Much as is evident among Aboriginal Australians in the historical period [115], foragers returning from hunting expeditions may have sometimes brought orphaned young Sulawesi warty pigs back to their home communities. This could have been undertaken with differing motivations. For example, some infant and juvenile *S. celebensis* pigs might have been given to small children as playthings, in which case, owing to rough handling, they are unlikely to have survived for long in human captivity. However, foragers would probably have also brought living some wild-caught piglets back to their homes with the intention of consuming them as food at a later stage, using them as live targets for instructing young children in the use of hunting weapons (e.g., [85]), and so on.

Once established on the island, AMH foragers probably would have occasionally brought back the captive young of an array of wild animal species encountered during hunting expeditions (e.g., birds, cuscuses, monkeys), not just *S. celebensis* (see, e.g., [46]). However, amid a general practice of keeping living wild juveniles of varying species as pets, it is proposed that a special relationship arose between foragers and *S. celebensis* pigs. This was owing simply to the fact that this was the largest game animal most frequently hunted by foragers, and hence humans had more opportunities to capture the living young of this species than other large mammal taxa, which, as noted, consisted only of anoas and babirusa. (It is assumed that the other endemic suid genus of Sulawesi, the archaic ‘giant’ pig *Celebochoerus*, known only from poorly dated fossil assemblages [144], was extinct before AMH colonisation). These suids are also considerably smaller in size than babirusa and anoas, so it seems likely they would have been easier for humans to manage even as adults. With an adult body weight of 40–85 kg [13], taxonomists and suid specialists variously describe *S. celebensis* as medium-sized [13,28], small in size, or very small ([27], p. 11). In comparison, adult babirusa weigh up to 100 kg [77], and anoas up to 150–300 kg [74]. Notably, modern miniature pig breeds that have gained popularity in some countries as exotic companion animals have an adult body weight of approximately 30 to 60 kg [129]. *S. celebensis* is broadly within the size range of these so-called minipigs or micropigs. Analysis of Toalean faunal assemblages also suggests that present-day *S. celebensis* is larger in size than the middle to late Holocene variant [145].

### 5.4. Foragers Would Have Taken Pre-Weaned Piglets from Wild Nests

AMH foragers would have known that the easiest way to obtain relatively large numbers of juvenile *S. celebensis* pigs was by raiding farrowing nests rather than tracking or ambushing sounders (social groups) or solitary mothers with dependent offspring. Targeting birthing places would have furnished whole litters of from 2–8 small, immobile, helpless individuals that were simple to capture compared with trying to seize hold of older, fast-running juveniles (though the latter is by no means impossible; e.g., [85], p. 214). In fact, it is plausible that foragers used their understanding of the reproductive biology of *S. celebensis* to time their foraging schedule in a manner that allowed them to predictably gain access to litters of pre-weaned Sulawesi warty piglets. In the western desert region of Australia, for instance, it is known from anthropological accounts that Pitjantjara people used astronomical cues to time their mass raids of wintertime dingo dens, taking the pre-weaned pups just as their eyes were opening ([146], p. 374). 

Recent findings also suggest that Late Pleistocene to middle Holocene hunter-gatherers inhabiting the tropical forests of montane New Guinea were strategically harvesting and managing the young of Dwarf cassowaries (*Casuarius* spp.) [147]. The rarity of cassowary bone in zooarchaeological assemblages indicates that foragers were not hunting these large (15–25 kg) flightless birds, but they did collect their eggs. Analysis of eggshell microstructural variation shows that foragers took the eggs from the nests (formed in shallow depressions in the ground) when they were in a late stage of embryonic development [147]. This suggests that people were utilising their knowledge of cassowary breeding seasonality in order to preferentially harvest eggs that were almost ready to hatch. The late-stage eggshells also showed few signs of burning, implying the early foragers were not cooking and consuming these eggs but instead eating the fully formed embryo (a delicacy in some present-day Asian-Pacific cultures) and/or allowing the eggs to hatch and rearing the chicks. Concerning the latter, the researchers note that ‘cassowary chicks imprint readily to humans and are easy to maintain and raise up to adult size’; indeed, human-raised cassowaries are a ‘traded commodity’ in parts of present-day New Guinea ([147], p. 8). Aboriginal foragers in the tropical north of eastern Australia also commonly reared wild-caught cassowary chicks and kept them as pets; for example, Donald Thomson observed that companion cassowaries were closely bonded to their adoptive human family, following people around the camp (and on their foraging rounds) like dogs [148]. The New Guinea data may provide the earliest known evidence for human management of the breeding of an avian taxon [147].

### 5.5. Foraging Women Would Have Suckled Pre-Weaned Piglets 

AMH foragers would have also discovered that keeping wild pre-weaned piglets alive for long enough to consume when they had grown larger required cross-species wet-nursing (i.e., breastfeeding by lactating women), without which the tiny youngsters would have swiftly weakened and died. Human breastmilk provides a vital source of nutrition-rich food for juvenile mammals. Lactation involves high energetic costs [149], but the burden of breastfeeding a small pre-weaned *S. celebensis* piglet is likely to have been relatively low. Wet-nursing young, adopted animals is a little different in principle to suckling another woman’s infant (allomaternal nursing), a widely documented practice among recent hunter-gatherer groups [150]. Foragers’ children are weaned at an average age of 2.5 years [151], although in some past foraging groups the cessation of breastfeeding did not occur until around 5–6 years [152]. An unweaned *S. celebensis* piglet could easily have been incorporated into such an extended infant feeding pattern. There is a plethora of ethnographic accounts and photographic records of women from nonindustrial societies—especially small-scale agriculturalists in Melanesia and Aboriginal foraging communities in Australia—nursing a human baby on one breast and a piglet (*S. scrofa*) or domestic dog (or wild-born dingo) puppy on the other [101,153]. The short period of time required to wean a *S. celebensis* piglet (several weeks, compared with years for a human infant) is unlikely to have greatly increased the metabolic demands of milk production for the lactating woman or caused nutritional deficits that affected the growth and development of her own child or others she was nursing. 

### 5.6. Keeping Wild-Caught Pigs as Pets Would Have Involved Human Selection

By rearing wild-born *S. celebensis* piglets in this way, it is likely that the foragers would have made some additional observations that were pertinent to human–pig companionship. First, they would have found that breastfed piglets often became closely bonded to their individual human caregivers and socialised with people within the wider family and community. That is, through humans intervening in the filial imprinting process, the piglets became tame animals that were habituated to the presence of humans and perceived them to be conspecifics. This is not a controversial supposition, being amply supported by modern ethological research and ethnographic studies of pig-rearing practices in New Guinea and wider Melanesia [132]. 

The difference between human-reared piglets and the piglets that had been captured in the wild after they had already been weaned by their mothers would have been known to foragers. Both were born in the wild, but the latter, having been reared by wild pigs, would have been closely bonded through the process of filial imprinting to members of their own species, not to humans. These older juveniles would have been wary of humans, their main predators. Most would have experienced visibly high levels of stress while living in captivity, shied away from any direct physical contact with humans, and used every opportunity to escape. It would have been necessary to keep these juveniles tethered or confined to a secure enclosure, cage, basket, or dwelling so they could not run away. It is likely to have been difficult to tame these older captives, and doing so would have required a long and laborious process of gradually habituating them to humans. Displays of affection towards these individuals are unlikely to have been reciprocated.

By contrast, foragers would have learned through trial and error that it was much easier to communicate and interact intimately with pre-weaned juveniles that had been taken from the wild and intensively nurtured by humans over an extended period of time. Unlike the case with weaned-in-the-wild piglets, it would have been possible for caregivers (and other community members) to form mutual bonds of attachment with most human-socialised piglets. In particular, once weaned off human milk, these juveniles most likely could have been trusted to wander freely around camp without the risk of them absconding. This is not to say that some individuals would not have reverted to the wild if they were left unrestrained; however, if a close pig–human bond was established through the nurturing process, then the possibility of pigs dispersing would have been reduced. In effect, most socialised pigs would not have wanted to leave the individual human(s) to which they were bonded. Chao [154] discusses the case of the Marind of West Papua and their penchant for keeping young wild-caught animals as pets. Disturbed by the quasi-human behaviour of these creatures, the Marind actively encourage them to return to the forest when they grow older. The animals become so attached to their adoptive human community, however, that they often steadfastly refuse to go [154].

Foragers also would have learned that, in a given litter taken from a wild nest, owing to individual variation in behavioural traits, some unweaned piglets would have been less easy to socialise than others and lacked other characteristics that humans tend to favour in companion animals (e.g., playfulness, tractability). This might also have included specific physical features favoured by humans for their aesthetic properties (e.g., novel pelage markings). Furthermore, as noted, some lactating women or other carers would have found that they became emotionally attached to some of the more sociable tame piglets they had breastfed and, or nurtured, played, and interacted with, and so on. In order to keep these young animals alive, they had effectively raised them as though they were their own children; inevitably, in some cases, they would have come to regard them as surrogate children, such that killing them was akin to murder and eating them to cannibalism. While some such individuals might still have been killed and consumed—they might have been reared by a lactating woman but owned by another community member—at least some might have been adopted as companions. (This was possibly how the first wild juvenile wolves came to live with hunter-gatherers of Late Pleistocene Eurasia: foragers took wild-born pups from spring dens and hand-raised them in order to slaughter them for their winter coat, with some individuals bonding closely to their caregivers during the rearing process and thus being spared and kept as pets [45]). By contrast, weaned *S. celebensis* juveniles taken from the wilderness could have been cared for by humans with the intention of eating them later, but it is unlikely these behaviourally wild youngsters would have been kept just for companionship.

### 5.7. Older Weaned Juvenile Pigs Would Not Be ‘Expensive’ Pets

Keeping a sexually immature pig as a pet in a forager community once it had been weaned would not have required much in the way of time and energy investments or the use of economic resources. Current evidence suggests *S. celebensis* is omnivorous, ‘with a wide-ranging diet reported to include roots, foliage, fallen fruits … bark, worms, insects, small vertebrates, and carrion’ ([13], p. 188). Hence, the natural feeding ecology of this species would have enabled it to readily adapt to the human niche as a scavenger that consumed food refuse—including inedible (for humans) components of wild plant foods (e.g., husks, fronds, leaves, root ends)—and human bodily excreta [41]. In fact, this service as a camp ‘cleaner’ could have been of benefit to foragers, keeping their living environment free of faeces and noisome food waste. (Dogs fulfil much the same function in many human communities, as does *S. scrofa*; e.g., [12], p. 83). Based on the ethnographic observations of pigs kept as pets in traditional societies in the region, it can be speculated that these favoured, socialised individuals perhaps would have been permitted to roam unrestrained around foraging communities as weaned juveniles and to forage without supervision in the vicinity of human encampments (which are unlikely to have been fenced). When these shifting groups of foragers moved to other locations, the young pigs probably would have followed the people to whom they were closely bonded and who provided their primary source of food. Carers might even have carried the small pigs on their journeys along with babies and small children. 

### 5.8. Human-Selected, Socialised Pigs Probably Would Have Bred near Humans

As the weaned individuals sexually matured, the human–pig dynamic is likely to have changed. Whether male or female, during the breeding season an adult socialised pig would have sought opportunities to mate. Being genetically wild, some young individuals of breeding age probably would have dispersed into the wild at this stage in their life history, their long-term bond with humans overridden by the immediate desire to mate (domesticated *S. scrofa* pigs often do the same, becoming ‘feral’). Others might have temporarily ‘escaped’ to breed, returning afterwards to their adoptive community with its reliable source of anthropogenic food. Assuming a given foraging group kept at least two socialised pigs of opposite sexes, some could have mated with each other either within the community or nearby. 

If a foraging community kept a solitary young, socialised pig as a pet, such individuals obviously would have been required to find a mate in the wild, with some returning after the breeding season and others not. In some cases, the socialised pigs that reverted to the wild when they reached sexual maturity may have established their territory near known sources of anthropogenic food, living in the wilderness close to where ‘their’ people camped. As with Pepina and ‘her’ wild pigs, foragers may have regarded these *S. celebensis* pigs living in their immediate area as companions or kin rather than potential prey, tolerating their presence, allowing them to visit their camps to scavenge food, and refraining from actively hunting or trapping them; indeed, Pepina’s rescue of Sahli suggests that living near humans might even have conferred a degree of protection from the pigs’ primary non-human predator, giant reticulated pythons, which have been observed to kill and eat adult *S. celebensis* pigs [155]—they are also known to eat adult humans [156]. Pigs that stayed close to foragers would have potentially mated with other human-socialised pigs and farrowed in nests located near foraging communities. 

Whatever the fate of the socialised adult pigs once they attained reproductive age, foragers would have acquired a new generation of wild-born, pre-weaned piglets once the gravid sows had birthed their young in farrowing nests. Hence, the process of rearing wild-caught piglets, some of which were eventually killed and consumed as food, and others adopted into the community as pets, would begin anew.

### 5.9. Keeping Pigs as Pets Might Have Altered Pig Populations in the Wild

If such a long-standing human–suid relationship had become established in one or more parts of Sulawesi, then the stage was set for wild populations of *S. celebensis* to undergo alterations in response. The scenario outlined here does not involve foragers asserting any level of conscious control over the reproductive processes of wild suids, selective breeding, or other forms of pig husbandry deemed essential for the domestication of a population of breeding adults [37]. The model involves foragers rearing wild-caught *S. celebensis* piglets that have been socialised to interact with humans (i.e., tamed wild animals), not genetically tame animals (those selectively bred for behavioural tameness)—the former constituting undomesticated animals, the latter (by most definitions) domesticated animals. Offspring born to tamed wild animals are not genetically tame, they are genetically wild, and hence the taming and socialisation process must be started afresh with each new generation [157]. However, if, as contended here, socialised wild pigs kept as pets by foragers returned to the wild to breed (or bred with other socialised wild pig companions), then it is possible that human selection could have begun to operate on free-ranging populations in the vicinity of foraging communities. As noted, human selection would have come into play right from the outset of foragers bringing a batch of living pre-weaned piglets into their home community: some individuals were kept alive owing to their particular favoured traits and others were culled. Hence, humans were selectively eliminating individuals from the local wild population that did not possess the traits that made them easy to tame and socialise, and promoting the survival of those that did. At least some of the individuals that survived into maturity were then introducing their genes into the wild genetic pool. 

If such a process did exist in the early history of Sulawesi, then it seems reasonable to infer that in parts of the island factors of geography and/or ecology may have enabled the effects of human selection to become more pronounced in wild-living *S. celebensis*. These were areas where foragers and wild suids were brought into close spatial proximity, such as in isolated patches of forest, montane valleys, or small satellite islands. In such settings, where humans and pigs inhabited small foraging ranges that overlapped significantly, regular dispersal of breeding-age socialised pigs from human communities could have exerted a more than random influence on standing genetic variation within the local population of purely wild pigs. Depending on how many socialised pigs the local foraging communities kept as pets, how often they returned to the wild to breed, and of course, how successful these pigs were at surviving and reproducing, we might expect to find an increase in the number of offspring born to these socialised individuals in the free-ranging pig population. If there were at least a few of these pets in any given community, and they bred in the wild successfully, then we might expect to find that more pigs in the wild population would inherit traits associated with tamability (and, or morphological characters favoured by humans for aesthetic reasons) that had been under human selection.

The effects of human selection might have also increased if human-reared *S. celebensis* pigs that had returned to the wild to breed and were accustomed to feeding and reproducing in proximity to humans became cut off from populations of solely wild-living *S. celebensis* pigs. This spatial division in ecotones might have occurred simply because wild unsocialised pigs were naturally fearful of humans and thus foraged and bred as far away from foraging communities, and their activity areas, as possible. Furthermore, foragers might also have engaged in the practice of tracking favoured socialised pigs to their birthing sites in the forest with the intent of obtaining their offspring, repeating the cycle when one or more of these erstwhile social companions produced offspring of their own; a practice documented among the Kubo of New Guinea in relation to wild-born feral sows hand-reared by women in villages [132]. If so, we might expect an inter-generational feedback loop to have operated, whereby the likelihood was increased that the *S. celebensis* piglets that foragers took from wild nests were the progeny of human-socialised pigs. Each time a new generation of wild piglets was introduced into a foraging community there was a higher probability that some individuals would have possessed traits that made them easier to tame and socialise. Genetic variation within the wild *S. celebensis* population from which individuals were selected to be tamed would be altered in response to the human preference for tamability, and the changes would become more pronounced, and the behaviours under selection more widespread in the local wild pigs, as time went on. This all could have occurred without foragers having any conception that their practice of adopting wild-born piglets as pets was changing the behaviour of the wild pigs in their environment.

### 5.10. If Domestication Occurred, It Might Have Been Linked to Animal Translocation

If there is any merit to Groves’s argument [14,16] that pre-Neolithic peoples domesticated *S. celebensis*, then it is probable that the causative factor in this major shift in human–suid relations would have been the human-mediated dispersal of human-socialised pigs from Sulawesi to other islands in the region. 

Once seafaring hunter-gatherers began taking *S. celebensis* pigs on ocean voyages populations of this wild suid would have become established in island habitats that lay well outside the species’ natural range. In these settings, socialised pigs could only have been able to breed with other pigs that, similar to them, had acquired traits that had been under a multigenerational process of indirect human selection (artificial selection) because they made them more amenable to taming and adoption as companion animals. In these genetically distinct, reproductively isolated populations, traits important to pet-keeping, such as tameness and sociability, might have been rife. Perhaps the most important trait under human selection would have been the increased propensity of socialised pigs to remain in their adoptive human communities after they had matured sexually rather than revert to the wild. If more socialised island pigs of breeding age reproduced within forager settlements instead of in the wilderness then the reproductive processes of the animals would effectively be under human control. 

At this stage, a transition to domestication may not have required the pig owners to begin living year-round in villages or domesticate wild plant foods. Contrary to conventional wisdom, even keeping a small group of adult *S. celebensis* pigs might not have required an agrarian economy to generate a supply of fodder if the pigs were allowed to consume food refuse and bodily waste around the camp and forage independently outside human communities; and, or, if, as some have proposed [20,91,92], foraging groups inhabiting the tropical forests of the region were intensively exploiting wild root and tree crops (e.g., banana, taro, sago, nuts), generating surplus food for pigs. It follows that if we are to look for evidence for the domestication of *S. celebensis* then we should probably not seek it on Sulawesi itself, but rather on other islands in Wallacea or the wider region where Groves believed pre-Neolithic peoples had introduced this species.

Seen from the perspective of *S. celebensis*, it may be the case that the innovation of sedentism and agriculture was not required to provision pigs per se but to develop the sort of complex social systems that allowed individuals to acquire large numbers of pigs that could be concentrated in one area (a village) and manipulated as a source of personal wealth and prestige. Domestication also presumably would have necessitated a shift in cultural attitudes towards socialised pigs. Instead of perceiving adopted pigs as being just companions, foragers had to be able to think of these human-reared animals as also comprising a source of meat. This shift in perception is likely to have required changes in the groups’ cultural philosophy and spiritual beliefs as they related to this particular human–animal relationship (see also [111,158]). 

## 6. Conclusions

This paper examined the long-standing argument proposed by Colin Groves that pre-agricultural foragers had domesticated the Sulawesi warty pig (*S. celebensis*) prior to the Neolithic farming transition in the region [14,16]. Groves’s contention is an accepted feature of the current specialist literature (e.g., [13]); however, it is as yet untested, and it has received little critical scrutiny from academics in the field. This is surprising, as the possibility that pre-Neolithic foragers independently domesticated a wild suid contravenes the prevailing model of initial pig domestication in Southwest Asia [11,37].

This paper’s focus has been on evaluating the plausibility of early domestication in *S. celebensis*. The perspective adopted here is that a pre-Neolithic population could have established a special relationship with *S. celebensis* that was based on inter-species companionship and cross-species adoption of wild-caught piglets; in essence, pet-keeping. It is argued that foraging groups might have routinely raided the farrowing nests of *S. celebensis* sows in order to obtain pre-weaned piglets, some of which were hand-reared and kept as socialised pets. Multigenerational histories of human–suid interactions based on pet-keeping could have instigated an evolutionary process wherein human selection for traits that made wild-caught piglets easier to socialise influenced variation within local wild pig populations. If so, early human interactions with *S. celebensis* involved a process of domestication that mirrors the so-called pet-keeping model for the Late Pleistocene domestication of dogs from wolves [45,46,47,48]. As noted previously, several scholars have critiqued the unrealistic and essentialist thinking behind combining Austronesian demographic expansion, sedentism, and agriculture (plus other Neolithic traits) into a single package [20,89,91]. Seen through this lens, the idea of a wholly indigenous and pre-Neolithic domestication of *S. celebensis* as originally postulated by Groves (albeit without further elaboration) is not as improbable as it might once have seemed. 

A great deal of archaeological data is obviously needed in order to test the possibility that pre-Neolithic people kept wild *S. celebensis* pigs as pets, and that unconscious selection causally linked to pet-keeping initiated further changes in human-associated populations of this wild suid. In particular, comprehensive zooarchaeological studies of pig remains from Holocene contexts in eastern Indonesia, especially Flores and Rote, including morphometric analyses and a focus on diet, along with captivity-related pathologies and proxies of human care, are required. In terms of archaeological evidence for early people keeping *S. celebensis* as pets, as with the earliest uncontested dogs [9], the ‘smoking gun’ would probably be the discovery of a forager buried together with one of these pigs (or just pig burials without humans), implying that the pig was a companion animal of some kind. Analyses of rock art representations of Sulawesi warty pigs might also prove fruitful [41]. In addition, genetic analyses of ancient *S. celebensis* remains may elucidate the presence of distinct human-associated lineages and uncover the pattern and timing of the human-mediated dispersals involving this suid (i.e., inter-island translocations). Future fieldwork and research will hopefully yield the empirical data needed to further develop and test the argument presented. In the meantime, the case of *S. celebensis* does suggest that the conventional wisdom that the domestication of a wild suid first required agriculture and sedentism is in need of reconsideration (see also [12], p. 62). This is an important point in the context of ancient Wallacea and Sahul, where there is evidence for the intentional translocation of wild animals [31] and management of birds (including the hatching and rearing of cassowary chicks [147]) prior to the Neolithic. 

Also in want of revision is the Eurocentric notion that early human relations with the wild ancestors of domestic pigs (and wild-living suids generally) were based solely on the exploitation of these animals as a source of meat, thus contrasting with the argument in which the wild progenitors of dogs were first brought into the domestic sphere as pets. This conception is clearly underpinned by the deep-seated image in Western thought of dogs as the archetypal human companion and the implicit view of pigs as farmed livestock rather than a contender for humanity’s ‘best friend’. The contention that dog domestication centred around the human adoption of wild-born wolf pups is compelling [45,46,47,48]. The overall story is more complicated, however, when viewed from the perspective of the less dog-centric cultures of the Asia-Pacific, where dogs and pigs can be both pets and food for humans. From this angle, pig domestication could have followed a wolf/dog-like pathway, especially in Sulawesi and other parts of the world where early foragers had a close and protracted relationship with wild suids.

## Data Availability

Not applicable.

## Appendix A. Claims of Pre-Neolithic Pigs, Including *S. celebensis*, on Flores

Apart from Sulawesi, it is only in Flores, from Liang Bua cave in the island’s west, that there is a standing claim for the presence of *S. celebensis* in a prehistoric context [24]. As some authorities have questioned this claim, especially the inferred date of 7 ka ([19], p. 262), it is worth examining the published evidence for it.

Larson and team [24] attempted to extract genetic material from a sample of 14 *Sus* teeth excavated from Holocene layers at Liang Bua. Of these, they reported that a total of seven teeth (N = 7) yielded mtDNA sequences attributed to a unique haplotype (GL550) ‘that clusters with modern Sulawesi *S. celebensis* samples’ ([24], p. 4837). These specimens came from excavation squares (Sectors) XI, IV, and III and ranged in depth from spit 6 (50–60 cm depth) to spit 29 (285–295 cm depth) (see SI Table 3 in [24]). The researchers only provided published age estimates for two specimens: a canine (Spec. #KD9675 & 9676) from spit 17 in Sector IV (160–170 cm depth), suggested to be older than around 4 ka, and a tooth (Spec. #LB/02) from spit 29 in Sector III. LB/02 was recovered nearly 3 m below the modern ground surface and is the deepest and oldest *Sus* tooth to have yielded *S. celebensis* mtDNA. The team contended that LB/02 dates to ~7 ka [24]; however, age inferences were based ‘on stratigraphic association and associated ^14^C dates of charcoal’ ([24], p. 4837) rather than directly dating teeth that yielded mtDNA.

The estimate of ~7 ka for the *S. celebensis* tooth seems questionable. There is only a single published ^14^C date (ANUA-23607) from the Holocene deposits excavated in Sector III. A charcoal sample recovered from a depth of around 363 cm yielded a mean calibrated age of 1,361 cal. BP [159]. According to Roberts et al. ([160], p. 493): ‘The latter sample has an anomalously young age (1–2 ka) for a depth of ~3.6 m, which we attribute to collection of the charcoal fragments from a deeply incised channel, evident in the east face of Sector III’. It is unclear whether the *S. celebensis* tooth (LB/02) came from this channel. However, LB/02 was recovered at least 65 cm above the location of the charcoal sample (ANUA-23607) dated to ~1.3 ka cal. BP [159], a provenance that does not build confidence in the inferred age estimate of ~7 ka. The original age of ~4 ka inferred for Spec. #KD9675 & 9676 from spit 17 in Sector IV (160–170 cm depth) seems to be more or less accurate based on the team’s recent major reassessment of the temporal sequence at Liang Bua [159,161]. With the exception of LB/02, however, all of the teeth that yielded *S. celebensis* mtDNA came from deposits dating to less than ~4 ka. It is possible that LB/02 was vertically displaced from more recent overlying contexts by disturbances associated with Neolithic or Metal Age pits or burials (G.D. van den Bergh, pers. comm. 2021).

It is worth noting that aDNA survival in faunal remains has been demonstrated at Liang Bua, but not as early as 7 ka. Evans et al. [162] analysed four excavated *Macaca* sp. (macaque monkey) bone and tooth specimens for aDNA. Direct-AMS ^14^C dating was conducted on all four specimens, yielding ages of 0.23–0.14, 1.53–1.40, 1.54–1.41, and 2.54–2.36 ka cal. BP, respectively. All of the specimens tested positive for aDNA, although only three had a sufficient amount of genetic material preserved for mitochondrial sequencing. The oldest of these has a calibrated mean age of 1.47 ka cal. BP [162].

Although it cannot be conclusively shown that any of the *Sus* teeth (N = 7) that yielded *S. celebensis* mtDNA have a genuinely pre-Neolithic antiquity, current evidence does seem to confirm that suids of some kind were present in Liang Bua during the accumulation of Unit 8A in the early to middle Holocene [159,163]. On the balance of the available evidence, therefore, it is reasonable to suggest that the human-mediated dispersal of *S. celebensis* to Flores occurred prior to the arrival of Austronesian-speaking populations in the region. Indeed, it seems unlikely (though not impossible) that ancient peoples would have translocated Sulawesi warty pigs between islands once domesticated *S. scrofa* swine (which tend to be larger and fatter than *S. celebensis*) were available.

### The Pig Fossil from the Sinkhole at Liang Bua

A recent investigation of a sinkhole that had formed below the main chamber at Liang Bua recovered a skull fragment from an unidentified pig species, and a carbonate coating on this bone yielded a Uranium-series age of 28 ± 5 ka [164]. The specimen (LB06-K-B1), dated to the Late Pleistocene, is described as comprising ‘the upper left orbit of a juvenile pig skull’ ([164], p. 542). Clearly, this claim presents several challenges. Given the context of the find—a Wallacean island for which there is no other evidence for endemic suiforms—the discovery that pigs of some kind were present in the Late Pleistocene is significant. However, the singular nature of the find is problematic: thus far, no fossils attributed to pigs have ever been found in the rich assemblages of Late Pleistocene faunal remains excavated over multiple seasons in the main chamber at Liang Bua [159,161,163,165]. There is no obvious reason to question the reliability of the U-series dating work carried out by this team on the mineral accretion that formed on the exterior surface of this bone. Their results suggest that whatever animal this skull fragment came from it died at least 23 ka ago and its remains were then incorporated into the sinkhole deposits at Liang Bua. The problem may therefore lie with the taxonomic assignation of this fragmentary specimen—that is, it is from some other juvenile mammal, not a pig. If the team has accurately identified this fossil, however—it is worth noting at this stage that Colin Groves was also a co-author on this paper—then the most plausible explanation would be that pre-Neolithic peoples used watercraft to introduce *S. celebensis* (or some other pig species) to Flores. If so, it would appear that this translocated suid taxon remained such a rare part of the island fauna on Flores that it is not represented in any of the faunal collections excavated thus far from the Late Pleistocene sediments in the main chamber at Liang Bua. Further analysis of the LB06-K-B1 specimen is clearly warranted.

There is credible evidence that seafaring hunter-gatherers were translocating at least some mammals between islands in the Wallacean archipelago. As noted, Late Pleistocene people apparently used watercraft to carry the northern common cuscus (*Phalanger orientalis*) from mainland New Guinea to New Ireland 23.5–20 ka ago [32]—this is among the oldest known record of an animal species dispersing through deliberate human agency [32]. There is no clear evidence for the human-mediated dispersal of larger animals such as pigs from island to island at such an early time in Wallacea or anywhere in the immediate region. The presence of babirusa as far east as Buru has, however, been taken as a possible indication of purposeful introduction by early humans [166].
